# Small-molecule and peptide inhibitors of m6A regulators

**DOI:** 10.3389/fonc.2025.1629864

**Published:** 2025-08-01

**Authors:** Xiaocui Liu, Xuefeng Kan

**Affiliations:** ^1^ Department of Radiology, Union Hospital, Tongji Medical College, Huazhong University of Science and Technology, Wuhan, China; ^2^ Hubei Provincial Clinical Research Center for Precision Radiology and Interventional Medicine, Wuhan, China; ^3^ Hubei Province Key Laboratory of Molecular Imaging, Wuhan, China

**Keywords:** RNA modification, N6-methyladenosine, inhibitors, molecular targets, cancer treatment

## Abstract

N6-methyladenosine (m6A) is a reversible mRNA modification that plays important roles in malignant tumor processes. m6A modification has emerged as a significant research focus. Studies on the functions and mechanisms of m6A and its regulatory factors across various tumors have grown increasingly comprehensive and in-depth. Accumulating evidence has demonstrated that m6A modifications and their associated regulatory proteins can serve as biomarkers for cancer treatment and prognosis. Consequently, there has been a surge in research on the development and application of m6A regulatory factor inhibitors, particularly regarding their efficacy and mechanisms in tumor therapy. These advancements not only enhance the understanding of their therapeutic potential in diverse cancers but also facilitate their integration with existing treatments, accelerating the design of more effective, specific, and selective inhibitors. Such efforts hold promise for advancing m6A-targeted pharmaceutical development and promoting clinical applications. This review summarizes small-molecule and peptide inhibitors of m6A regulators for malignant tumors.

## 1 Introduction

N6-methyladenosine (m6A) specifically refers to the methylation occurring at the nitrogen-6 position of adenine, which is one of RNA modifications. This modification predominantly targets the conserved RRACH sequence (R = A/G, H = A/C/U) and is mainly enriched near the termination codon and 3’ untranslated region (3’UTR) (1, 2). RNA m6A methylation involves modifications in both coding mRNAs and non-coding RNAs (ncRNAs), with the latter category encompassing constitutive ncRNAs and regulatory ncRNAs (3–8). M6A influences the fate of modified RNA molecules by altering mRNA structure, maturation, stability, splicing, transport, localization, translation, degradation (9, 10), and the processing of miRNAs (11) or lncRNAs (12), as well as mediating RNA-protein interactions (13). In eukaryotes, m6A is the most abundant and conserved form of mRNA methylation modification (14). First discovered in mammalian mRNAs in 1974 (15), m6A has become a central focus of RNA epigenetics research, particularly with the advent of advanced sequencing technologies. These advancements have established m6A as the most extensively studied RNA methylation modification, especially in the context of cancer, where research on m6A modification of mRNAs has gained significant momentum (16).

m6A has a widespread impact on the normal physiological and biochemical processes of the body, as well as the occurrence and development of diseases, including metabolism, growth and development, viral infections, chronic diseases, and tumors (17–26). m6A and its regulatory proteins have emerged as critical diagnostic, therapeutic and prognostic biomarkers for cancers. Abnormal m6A modification levels are implicated in the initiation and development of various tumors in multiple systems throughout the body. This post-transcriptional modification exerts either oncogenic or tumor-suppressive effects by modulating m6A levels in mRNAs of involved genes, as well as by activating downstream signaling pathways (27). Aberrant expression of m6A regulators promotes growth, proliferation, migration, invasion, and metastasis of malignancies through multiple mechanisms (28–30). These include inducing tumor cell cycle arrest, inhibiting apoptosis and differentiation, disrupting cancer stem cell self-renewal, altering the tumor microenvironment, and enhancing immune evasion, all of which contribute to tumorigenesis and progression (31–36). Furthermore, the abnormality not only leads to the occurrence of radiotherapy or chemotherapy resistance (37–39), but also devastates the effectiveness of immunotherapy (40–43), ultimately influencing patient survival and prognosis.

The roles and mechanisms of m6A-related regulators in various tumors have been extensively studied and systematically summarized. In recent years, numerous studies have highlighted the potential of inhibitors targeting m6A regulatory proteins, offering promising benefits for cancer treatment. Despite these advancements, a comprehensive review specifically addressing the research progress of m6A inhibitors in cancer has been lacking. This review seeks to provide a comprehensive summary of the current research on the efficacy and mechanisms of all m6A regulatory factor inhibitors with demonstrated cellular activity in tumors. It aims to serve as a valuable reference for expanding the application of m6A-targeted inhibitors, integrating them with existing therapeutic strategies, and overcoming resistance to chemotherapy. Furthermore, this review describes the potential for m6A-targeted pharmaceuticals to cooperate with new materials and convert into clinical practice, offering new prospects for the treatment of incurable cancers.

## 2 Overview of m6A methylation regulatory factor

**Figure 1:**
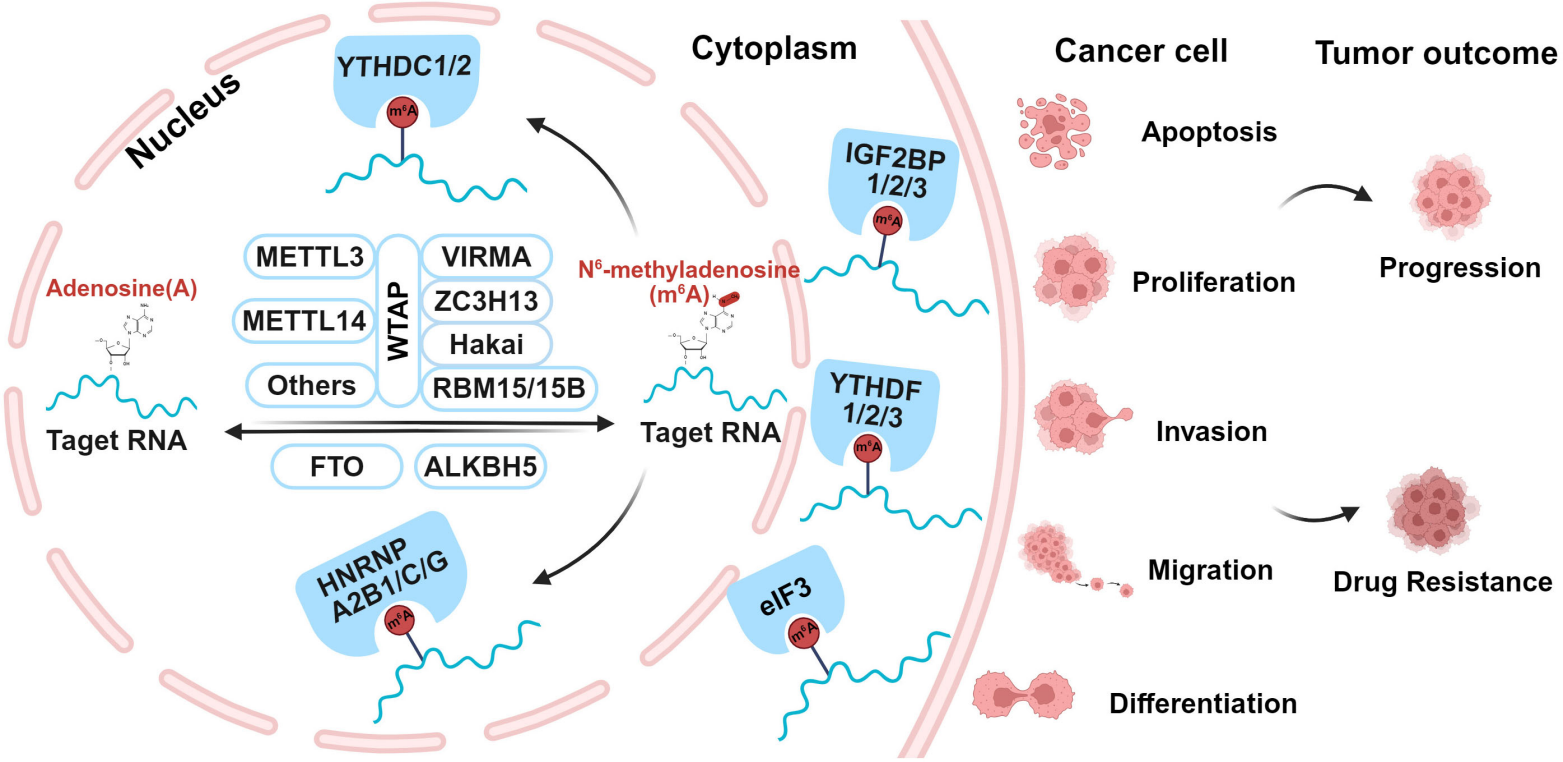
Schematic diagram of the mechanism and function of m6A and its regulatory proteins.

m6A modification is a dynamic and reversible process, which is regulated by three distinct types of proteins, including Writers, Erasers, and Readers, as shown in **Figure 1**. Writers are m6A methyltransferases that responsible for catalyzing RNA methylation; Erasers are m6A demethylases that remove methyl groups from RNA; Readers are a group of m6A-specific binding proteins that selectively recognize and interpret m6A modifications (40, 44–46).

### 2.1 m6A methyltransferase

The m6A methyltransferases, known as “writers”, can be classified into three major categories: methyltransferase-like 3 (METTL3), methyltransferase-like 14 (METTL14), and Wilms tumor 1-associated protein (WTAP). Other members include METTL5/16, RBM15, RBM15B, ZC3H13, ZCCHC4, CBLL1, VIRMA, and Hakai (47). Together, these proteins form the N6-methyltransferase complex (MTC), which utilizes S-adenosylmethionine (SAM) as a methyl donor to catalyze m6A methylation at the nitrogen-6 position of adenine in RNA (48). The MTC operates through the coordinated interaction of two core proteins, METTL3 and METTL14. They form a stable METTL3/14 heterodimer, serving as the catalytic core of the complex, while additional regulatory subunits provide structural support and fine-tune its activity (49). Both METTL3 and METTL14 belong to the S-adenosyl-L-methionine-dependent methyltransferase superfamily and bind either SAM or S-adenosylhomocysteine at the catalytic site. METTL3 contains an activated methyltransferase structural domain, which serves as the catalytic core to transfer the methyl group from SAM to adenosine (50). This domain is capable of catalyzing m6A modification of mRNA. In contrast, METTL14 lacks catalytic activity but specifically facilitates the recognition of METTL3 and the binding of MTC to the RNA substrates, thereby enhancing the catalytic activity of METTL3 (51). WTAP integrates and interacts with the METTL3/14 complex, regulating the localization of the MTC by recruiting it to nuclear speckles enriched with pre-mRNA processing factors. It maintains the catalytic activity of m6A methyltransferase, and also adjusts the recruitment of MTC to target mRNAs (52). Although METTL3, METTL14, and WTAP constitute the major components of the MTC, the contributions of other members should not be overlooked. VIRMA induces regioselective methylation by recruiting the catalytic major components, and mediates preferential methylation of mRNA (53). ZC3H13 regulates and promotes nuclear localization of MTC by facilitating nuclear translocation of WTAP, VIRMA and Hakai (54). Additionally, it serves a regulatory role as a bridge between RBM15 and WTAP (55). RBM15/15B interacts with the MTC and guides its recruitment to specific RNA sites, facilitating the deposition of m6A modifications (56). Hakai stabilizes the major components of MTC (57). These activities illustrate the diverse roles of m6A methyltransferase with diverse components in RNA regulation and cellular processes.

### 2.2 m6A demethylase

The m6A demethylases, also called “ Erasers “, include fat mass and obesity-associated protein (FTO) and alkB homolog 5 (ALKBH5), both of which belong to the ALKB protein family of Fe^2+^/α-ketoglutarate-dependent dioxygenases (58). They remove m6A modifications by catalyzing the hydroxylation of their target bases, thereby reducing RNA methylation levels and effectively reversing the methylation process (58, 59). FTO oxidizes m6A to unstable intermediates, including N6-hydroxy-methyl-adenosine (hm6A) and N6-formyl-adenosine (f6A), ultimately converting them into normal adenosine (A), thereby carrying out its demethylation function (60, 61). FTO primarily targets pre-mRNAs, influencing alternative splicing and 3’UTR processing (62). Though FTO can demethylate both mRNA m6A and cap m6Am in the cytoplasm, its primary demethylation activity occurs in the nucleus. In contrast, ALKBH5 localizes to nuclear speckles and specifically demethylates nuclear RNA substrates (63, 64). It directly converts m6A to A and catalyzes the release of f6A via the R130/K132/Y139 triad (60). ALKBH5-mediated erasure of m6A is required for proper splicing and the generation of longer 3’UTR mRNAs (65), ultimately affecting overall mRNA output (66). These activities underscore the critical roles of FTO and ALKBH5 in regulating RNA methylation dynamics and their subsequent impact on RNA metabolism.

### 2.3 m6A methylation recognition proteins

Readers, also referred to as methylation effectors or methylated reading proteins, specifically recognize and bind to m6A modifications. This diverse group primarily includes the YT521-B homology (YTH) domain family, insulin-like growth factor 2 mRNA-binding proteins (IGF2BPs), HNRNPs, and eIF3 (67). Additionally, it encompasses BP1, G3BP2, PRRC2A, RBMX, FMR1, ELAVL1, G3BP1, and G3BP2 (68). Among them, YTH domain family consists of YTH domain family proteins 1–3 (YTHDF1–3) and YTH domain-containing protein 1–2 (YTHDC1–2). IGF2BPs are made up of IGF2BP1-3, also known as IMP1-3 (69). Meanwhile, HNRNPs encompass HNRNPC, HNRNPG, and HNRNPA2B1. Readers recognize m6A-modified bases through distinct regulatory mechanisms and subsequently bind to m6A-containing mRNAs, influencing their fate. By selectively recognizing m6A modification sites, these proteins play various roles in regulating key RNA processes, including splicing, translation, transport, and degradation. YTHDF1–3 specifically recognize m6A-modified mRNAs primarily in the cytoplasm. YTHDF1 induces the further translation of m6A-labeled transcripts into proteins (70–72), while YTHDF2 leads to mRNA decay (73, 74). YTHDF3 exhibits versatile roles in regulating RNA processing, translation and decay, with its binding to YTHDF1 to promote translation and YTHDF2 to facilitate degradation (75, 76). Unlike YTHDF1-3, YTHDC1–2 primarily function in the nucleus. YTHDC1 is involved in modulating mRNA selective splicing (77, 78), mediating mRNA nuclear export (79), accelerating the decline of certain transcripts (80). YTHDC2 contributes to mRNA stabilization and translation (81, 82). The IGF2BP1–3 proteins stabilize the target mRNAs and enhance their translation efficiency (83). HNRNPC and HNRNPG primarily interact with ncRNAs and also bind m6A-modified mRNAs, facilitating their splicing (45, 84). HNRNPA2B1 benefits the maturation, transport, and metabolism of mRNA, as well as regulating lncRNA expression (85, 86). The eIF3 protein binds to m6A modification sites at the 5’UTR of mRNA, facilitating translation through a novel mechanism of eIF3-driven initiation that operates independently of cap-binding factors (87). All above highlight the diverse regulatory roles of m6A readers in RNA biology and their impact on cellular processes.

## 3 METTL3 inhibitors

### 3.1 Advancements in classification, mechanisms, and therapeutic potential

Research on m6A methyltransferase regulatory factor inhibitors, as shown in **Table 1**, has predominantly centered on the core protein, METTL3. From one perspective, METTL3 inhibitors can be categorized into nucleoside and non-nucleoside compounds, with the latter further subdivided into allosteric and competitive inhibitors. Nucleoside compounds, mainly comprising adenosine and its analogs, are characterized by their poor selectivity, like neofenobufenacin (88). The allosteric inhibitor CDIBA, which features an indole core structure, binds non-competitively and reversibly to the METTL3-METTL14 enzyme complex, and compound 43n optimized from CDIBA exhibits significant antiproliferative efficacy against acute myeloid leukemia (AML) cells (89). However, an increasing number of studies are now focusing on competitive inhibitors.

**Table 1:** Summary of m6A methyltransferase inhibitors.

Targets	Inhibitors	Types	Cancers	Research types		Ref.
METTL3	compound 43n	Synthetic compound	AML	*In vitro*	NA	(89)
	quercetin	Natural product	PCa	*In vitro*	NA	(91)
			HCC	*In vitro*	NA	(91)
	Lobeline	Natural product	HCC	*In vitro*	*In vivo*	(92)
	UZH1a	Synthetic compound	AML	*In vitro*	NA	(94)
			OS	*In vitro*	NA	(94)
	UZH2	Synthetic compound	AML	*In vitro*	NA	(95)
			PCa	*In vitro*	NA	(95)
	STM2457	Synthetic compound	AML	*In vitro*	*In vivo*	(96, 103–106)
			NSCLC	*In vitro*	*In vivo*	(97, 107–109)
			SCLC	*In vitro*	*In vivo*	(112)
			ICC	*In vitro*	*In vivo*	(113)
			HCC	*In vitro*	*In vivo*	(114–116)
			RCC	*In vitro*	*In vivo*	(117–119)
			NB	*In vitro*	*In vivo*	(120, 121)
			SHH-MB	*In vitro*	*In vivo*	(122)
			OS	*In vitro*	*In vivo*	(123)
			CRC	*In vitro*	*In vivo*	(124–126)
			OSCC	*In vitro*	*In vivo*	(127)
			BC	*In vitro*	*In vivo*	(128, 129)
			PCa	*In vitro*	*In vivo*	(130)
	STC-115	STM2457 derivative		Clinical trial		(90)
	compound C3	Synthetic compound	NA	NA	NA	(98)
	RMS	peptide inhibitor	PCa	*In vitro*	*In vivo*	(100)
			melanoma	*In vitro*	*In vivo*	(101)
	RSM3	peptide inhibitor	PCa	*In vitro*	*In vivo*	(99, 100)
			AML	*In vitro*	NA	(99)
			NSCLC	*In vitro*	NA	(99)
	EP652	Synthetic compound	AML	*In vitro*	*In vivo*	(102)
			OC	*In vitro*	*In vivo*	(102)
			NSCLC	*In vitro*	*In vivo*	(102)
			OSCC	*In vitro*	*In vivo*	(102)
METTL14	WKYMVM	Synthetic compound	BC	*In vitro*	*In vivo*	(131)

AML, Acute Myeloid Leukemia; PCa, Prostate Cancer; HCC, Hepatocellular Carcinoma; OS, Osteosarcoma; NSCLC, Non-Small Cell Lung Cancer; SCLC, Small Cell Lung Cancer; ICC, Intrahepatic Cholangiocarcinoma; RCC, Renal Cell Carcinoma; NB, Neuroblastoma; SHH-MB, Sonic Hedgehog Medulloblastoma; CRC, Colorectal Cancer; OSCC, Oral Squamous Cell Carcinoma; BC, Breast Cancer; OC, Ovarian Cancer.

From a complementary perspective, investigations into METTL3 inhibitors have primarily concentrated on four key domains: the application of natural pharmaceutical compounds, clinical evaluation of synthetic small-molecule drugs, the amalgamation of extensive datasets with computational predictive modeling, and the identification of drug targets and associated pathways (90). Quercetin, a natural compound, has been recognized as the pioneering METTL3 inhibitor. Studies showed that it exerts anti-proliferative effects by reducing global m6A levels in prostate cancer (PCa) and hepatocellular carcinoma (HCC) cell lines (91). Lobeline, another naturally derived inhibitor, was initially identified through screening of a natural product library (92). Research demonstrated that lobeline significantly enhances HCC chemosensitivity to Lenvatinib and effectively reverses therapeutic resistance, resulting in tumor inhibition (92). Mechanistically, it is linked to the regulation of UBE3B expression and function (92). Moreover, studies confirmed that many natural drug components regulating m6A methylation activity fall into categories like polyphenols, flavonoids, alkaloids, anthraquinones, and terpenoids (93).

In a series of studies involving protein crystallography, biochemical binding assays, and cellular experiments, Elena V. et al. (94) identified and characterized UZH1a, a small-molecule METTL3 inhibitor with selectivity, cell permeability, and potency. It effectively reduces the mRNA m6A/A ratio in various cell lines, including osteosarcoma and AML cells. Building on this, Dolbois et al. optimized a METTL3 hit compound using medicinal chemistry and protein crystallography techniques, ultimately leading to the development of lead compound UZH2, and it specifically inhibits intracellular METTL3 activity and significantly decreases the m6A levels in AML and PCa cell lines (95). STM2457 represents a breakthrough in METTL3 inhibition as the first bioavailable compound. This highly selective and potent inhibitor demonstrates both cellular activity and oral bioavailability, making it the most extensively characterized and clinically promising METTL3 inhibitor to date (96). STM2457 (C_25_H_28_N_6_O_2_; MW: 444.53 g/mol; CAS No. 2499663-01-1) is usually supplied as a stable solid powder (97). Since its initial discovery, it has undergone comprehensive pharmacological evaluation, exhibiting potent METTL3-targeting efficacy across various solid and hematological tumors. The STM2457 derivative STC-15 is the first clinical candidate oral drug targeting METTL3. Currently, a Phase I clinical trial (NCT05584111) is underway to assess STC-15 in patients with advanced solid tumors (90). This marks a major milestone in targeted cancer therapy with the development of METTL3-specific inhibitors as a new therapeutic strategy.

Additionally, Yang et al. (98) employed a series of rigorous and sophisticated methods to screen and identify the compound 2-(4-hydroxyphenyl)-5-[3-[2-(4-hydroxyphenyl)-1,3-dioxoisoindol-5-yl] oxyphenoxy] isoindole-1,3-dione (C3). which exhibits potent and selective inhibitory activity against METTL3. Though its efficacy has not yet to be further validated through preclinical studies, this discovery provides a novel perspective for the development of METTL3 inhibitors.

Unlike small molecule inhibitors, peptide inhibitors were innovatively developed. The representative peptide inhibitor RM3 exhibits significant antitumor properties by inhibiting METTL3 activity and promoting its degradation (99). Based on this, a stable peptide inhibitor named RSM3 was designed and confirmed its antitumor efficacy in various malignancies, including PCa, AML, and NSCLC (99). Furthermore, RM3 and RSM3 effectively inhibit PCa progression, where METTL3/m6A/RRBP1 plays a promoting role (100). Similarly, RM3 exerts anti-tumor effects on melanoma cells (101). Moreover, combining RM3 with anti-PD-1 shows a comparably greater anti-tumor efficacy (101). These findings lay the foundation for developing small peptide inhibitors of METTL3 and also provide new cancer therapy for binding peptide-based drugs with immune checkpoint inhibitors (ICIs).

Recently, Dutheuil et al. (102) discovered and optimized a new effective METTL3 small molecule inhibitor, EP652. In *in vivo* models of AML and ovarian cancer (OC), EP652 significantly inhibited cancer progression. This discovery opens new avenues for therapeutic strategies in both solid and hematologic tumors.

### 3.2 Applications of METTL3 inhibitor STM2457 in cancers

#### 3.2.1 Acute myeloid leukemia

METTL3 serves as an oncogene in AML, thereby promoting AML progression (103, 104). Two primary mechanisms have been identified (1): Hyperactivation of METTL3 evokes AML cell proliferation and inhibits differentiation by promoting the translation of oncogenic targets such as c-MYC, BCL2, and PTEN (103); (2) CAATT enhancer binding protein Zeta (CEBPZ) recruits METTL3 to the transcription start site, leading to the enhanced translation of oncogenes SP1 and SP2 (104). Yankova et al. indicated that STM2457 lessened AML growth rate and induced differentiation and apoptosis, resulting in anti-leukemic outcomes (96). Similarly, Li et al. demonstrated that METTL3 can promote m6A modification of ITGA4 mRNA, thereby enhancing the mRNA stability and prolonging its half-life (105). This leads to the upregulation of ITGA4 protein expression, which subsequently promotes the homing and implantation of AML cells. Furthermore, AML cells may interact with the bone marrow microenvironment and finally provoke chemoresistance in AML (105, 106). However, STM2457 can significantly turn around the homing and implantation of AML cells, thus reversing drug resistance and improving efficacy (105). These findings highlight the potential of STM2457 as a promising therapeutic agent in combating AML and overcoming chemoresistance.

#### 3.2.2 Lung cancer

The heterogeneity of non-small cell lung cancer (NSCLC) promotes its drug resistance, and PD-L1 expression suppresses its immunotherapy. STM2457 effectively inhibits the progression of NSCLC by targeting the translational regulatory mechanisms of METTL3 and its interplay with PD-L1 (97). STM2457 not only overcomes the heterogeneity of tumors to achieve inhibition of tumor progression, but also regulates the expression of PD-L1, thereby expanding the benefits of immunotherapy. Additionally, STM2457 can inhibit the METTL3/m6A/SRPK1 axis, thereby preventing glycolysis in lung adenocarcinoma (107). Generally speaking, METTL3 promotes glycolysis via increasing the m6A modification of SRPK1, thereby facilitating tumor progression. Furthermore, the combination of STM2457 and MAT2A inhibitors displays good synergistic anti-tumor effectiveness (108). Notably, the METTL3 inhibitor specifically targets the PI3K/AKT signaling axis. Additionally, the combination of STM2457 with paclitaxel (PTX) or carboplatin demonstrates significantly enhanced synergistic anti-tumor efficacy in NSCLC (109). The mechanism involves the reduction of ABCC2 expression in an m6A-YTHDF1-dependent manner.

In contrast to NSCLC, small cell lung cancer (SCLC) presents formidable therapeutic challenges characterized by its aggressive biological behavior, high malignant potential, and drug-resistant susceptibility (110, 111). The elevated expression of METTL3 mRNA and protein levels in SCLC individuals predicts poor prognosis (112). METTL3 reduces the m6A level of DCP2 mRNA, accelerating its degradation and suppressing DCP2 expression (112). This process activates Pink1-Parkin-mediated mitochondrial autophagy, maintaining cellular homeostasis and promoting chemotherapy resistance in SCLC (112). These findings highlight STM2457’s potential to target similar mechanisms in both NSCLC and SCLC, offering a promising strategy to overcome drug resistance and improve treatment outcomes in these aggressive lung cancers.

#### 3.2.3 Intrahepatic cholangiocarcinoma

The overexpression of METTL3 in intrahepatic cholangiocarcinoma (ICC) patients is associated with poor prognosis (113). The upregulation of METTL3 promotes m6A modification of IFIT2 mRNA, reducing its stability and triggering its degradation. This leads to the downregulation of IFIT2 expression, which in turn facilitates tumor progression (113). However, the METTL3 inhibitor STM2457 effectively suppresses ICC by inhibiting cell proliferation, migration, and invasion, as well as inducing apoptosis in ICC cells (113). These findings show the potential of STM2457 in improving ICC patient treatment outcomes.

#### 3.2.4 Hepatocellular carcinoma

METTL3 overexpression is strongly linked to poor prognosis and resistance to oxaliplatin (OXA) in HCC individuals (114). Mechanistically, the METTL3/TRIM21/G6PD pathway works well in promoting drug resistance of HCC (114). Notably, STM2457 reverses the drug resistance and enhances the therapeutic efficacy in HCC (114). In another study, abundant m6A was discovered in the core enzymes of SSP (PSPH, PSAT1, and PHGDH), and the METTL3/m6A/IGF2BP3 axis works in modulating associated mRNA stability and activating SSP (115). Furthermore, HCC cells with resistance to sorafenib depend on m6A to regulate the SSP pathway and reactive oxygen species (ROS) levels (115). Importantly, inhibiting SSP with STM2457 increases ROS accumulation, impairs HCC growth, and enhances the sensitivity of HCC to sorafenib (115). Additionally, STM2457 targets the METTL3/m6A/BMI1/RNF2 signaling pathway to suppress malignant growth (116). These studies provide more promising therapeutic ideas for the treatment of HCC.

#### 3.2.5 Renal cell carcinoma

Elevated METTL3 expression in advanced RCC patients equals to poor prognosis (117). RCC tends to generate a hypoxic microenvironment due to rapid growth (118). Hypoxia upregulates METTL3 via HIF-1α, promoting m6A-modified PLOD2 mRNA translation, ultimately resulting in RCC migration and invasion (117, 119). Significantly, STM2457 targets the METTL3-PLOD2 axis, effectively reducing tumor growth and metastasis. These findings highlight STM2457 as a promising therapeutic agent for advanced RCC.

#### 3.2.6 Neuroblastoma

On the one hand, STM2457 inhibits the proliferation of adrenal neuroblastoma and promotes a differentiated phenotype, subsequently restraining neuroblastoma growth (120). Additionally, the study preliminarily explored the role of differentiation-related gene expression in this process. On the other hand, KAP1, in conjunction with YTHDC1 and METTL3, forms a complex responsible for preserving the stability of MYCN mRNA (121). Consequently, STM2457 downregulates MYCN to achieve an anti-tumor effect in neuroblastoma. These findings provide a foundation for further mechanistic investigations and the development of potential therapeutic methods.

#### 3.2.7 Sonic hedgehog medulloblastoma

High expression of METTL3 means poor prognosis in patients with Sonic Hedgehog medulloblastoma (SHH-MB) (122). The over-expression of METTL3 leads to persistent m6A hypermethylation of PTCH1 and GLI2, thereby promoting proliferation and suppressing cell death (122). However, STM2457 inhibits METTL3, thereby reducing tumor progression. Which presents a promising therapy for SHH-MB.

#### 3.2.8 Osteosarcoma

Elevated expression of METTL3 incurs an increase in the overall m6A modification level of the oncogene ZBTB7C mRNA, eventually promoting osteosarcoma progression (123). Notably, STM2457 decreases m6A modification levels and weaken ZBTB7C protein abundance, leading to osteosarcoma growth inhibition. Furthermore, the increased METTL3 and ZBTB7C levels in human osteosarcoma tissues were confirmed, highlighting their potential as therapeutic targets and STM2457 as a promising drug.

#### 3.2.9 Colorectal cancer

Yu et al. (124) discovered that STM2457 exhibits significant anti-tumor activity in CRC cells and tissues. Mechanistically, the inhibition of METTL3 reduces the m6A level of asparagine synthetase (ASNS) mRNA, thereby downregulating ASNS expression and exerting anti-tumor effects. However, Zhou et al. (125) found that m6A modification enhances mitochondrial fusion via RRM2B/GSH/OPA1 pathway, and STM2457 prevent the above fusion in order to prohibit CRC progression. Additionally, the combination of STM2457 with an anti-PD-1 antibody can strengthen the expression of interferon-gamma and granzyme B, thereby increasing the cytotoxic activity of T cells and boosting the anti-tumor immunity against colon cancer, as well as melanoma (126). These studies offer novel insights into combination therapy.

#### 3.2.10 Oral squamous cell carcinoma

STM2457 combined with anlotinib could effectively prevent OSCC (127). Mechanistically, this combination significantly downregulates the expression of epidermal growth factor receptor (EGFR), suppresses the stemness properties and epithelial-mesenchymal transition (EMT) process. This combined therapeutic remedy may bring new insights for the clinically treatment of OSCC.

#### 3.2.11 Breast cancer

A study (128) revealed a progression mechanism in triple-negative breast cancer (TNBC), where POP1 targets m6A to destabilize CDKN1A mRNA. The downregulation of CDKN1A accelerates TNBC progression. Notably, STM2457 not only reverses this situation but also increases the sensitivity of TNBC to PTX. Another study (129) about the endoplasmic reticulum (ER) discovered a new mechanism where XBP1s enhances the expression of METTL3/METTL14 and increases its m6A modification. In turn, the elevated m6A modification facilitates reticulophagy (ER-phagy) by stabilizing the mRNA of CALCOCO1 and p62, ultimately supporting cell survival under PTX-induced stress in BC. Further investigation showed that PTX could mediate ER stress and up-regulate m6A modification for ER-phagy. Importantly, experiments on BC declared remarkable therapeutic efficacy through combining STM2457 and PTX. These evidences bring novel approaches for PTX resistance in BC.

#### 3.2.12 Prostate cancer

Recent studies (130) highlighted the therapeutic potential of targeting m6A modifications in PCa. Notably, STM2457 effectively reduces m6A levels in PCa cells, thereby impairing their proliferative, invasive, migratory, and stemness properties. Moreover, STM2457 demonstrates significant tumor-suppressive effects in animal models. Mechanistically, the IGFBP3/AKT axis acts as a key mediator of its biological effects. Additionally, STM2457 exhibits synergistic anti-PCa activity when combined with the PARP inhibitor olaparib. Undoubtedly, these findings provide new insights into the treatment and combination therapy of PCa.

## 4 METTL14 inhibitors

As one of the core components of methyltransferase, there have been few reports on METTL14 inhibitors. Recently, Liu et al. (131) identified a novel small molecule inhibitor, WKYMVM (Trp-LysTyr-Met-Val-Met-NH2), through screening. The study showed that METTL14 can increase the expression of E2F1 in BC, thereby promoting its resistance to CDK4/6 inhibitor therapy. However, the inhibitor WKYMVM can reverse the resistance of BC cells to CDK4/6 inhibitor treatment, restoring their sensitivity. This breakthrough paves the way for the development and exploration of METTL4 inhibitors.

## 5 FTO inhibitors

All demethylase inhibitors are shown in **Table 2**. FTO was the first RNA m6A demethylase to be discovered (64). To date, more than 10 FTO inhibitors have been identified, and the therapeutic efficacy of these inhibitors has been evaluated across various cancers. FTO inhibitors can be classified into three distinct categories based on their mechanism of action (1): metal ion-chelating inhibitors that disrupt the catalytic center, (2) 2-oxoglutarate(2-OG) analogs that compete with the essential cofactor, and (3) selective m6A-competitive inhibitors that specifically target the substrate-binding site (132). Targeting FTO can suppress tumor growth, enhance tumor immunotherapy, and mitigate tumor drug resistance (32). The development of FTO inhibitors provides additional therapeutic solutions for cancer treatment.

**Table 2:** Summary of m6A demethylase inhibitors.

Targets	Inhibitors	Types	Cancers	Research types		Ref.
FTO	Rhein	Natural product	AML	*In vitro*	*In vivo*	(137–139)
			BC	*In vitro*	*In vivo*	(140–142)
	SsD	Natural product	AML	*In vitro*	*In vivo*	(143)
	Mupirocin	Natural product	CRC	*In vitro*	*In vivo*	(144)
	MO-I-500	Synthetic compound	BC	*In vitro*	*In vivo*	(145–147)
	MA	Synthetic compound	AML	*In vitro*	*In vivo*	(138)
		bladder cancer		*In vitro*	NA	(149)
			BC	*In vitro*	NA	(149)
			NSCLC	*In vitro*	*In vivo*	(149–153)
	MA2	Synthetic compound	GBM	*In vitro*	*In vivo*	(154, 155)
	compound 11b	Hz-MA analogs	AMOL	*In vitro*	NA	(156)
	FB23/FB23-2	MA derivative	AML	*In vitro*	*In vivo*	(157–159)
			CC	*In vitro*	NA	(160)
			ccRCC	*In vitro*	*In vivo*	(161)
	13a	FB23 derivative	AML	*In vitro*	*In vivo*	(162)
	44/ZLD115	FB23 derivative	AML	*In vitro*	*In vivo*	(163)
	Dac51	FB23 derivative	melanoma	*In vitro*	*In vivo*	(164)
	FTO-04	Synthetic compound	GBM	*In vitro*	*In vivo*	(165)
	FTO-43N	FTO-04 derivative	GC	*In vitro*	*In vivo*	(166)
	R-2HG	2-OG analogs	AML	*In vitro*	*In vivo*	(167)
	CS1, CS2	Synthetic compound	AML	*In vitro*	*In vivo*	(158)
			CRC	*In vitro*	*In vivo*	(169)
			PCa	*In vitro*	*In vivo*	(170)
	18097	Synthetic compound	BC	*In vitro*	*In vivo*	(171)
	compound C6	Synthetic compound	EC	*In vitro*	*In vivo*	(172–175)
	compound 8t	Synthetic compound	AML	*In vitro*	*In vivo*	(176)
	2,4-PDCA	2-OG analogs	NA	NA	NA	(132)
	IOX1	2-OG analogs	NA	NA	NA	(132)
	FL6, FL8	fluorescein analogs	CC	*In vitro*	NA	(133)
	NCDPCB	Synthetic compound	NA	NA	NA	(134)
	CHTB	Synthetic compound	NA	NA	NA	(135)
	radicicol	Natural product	NA	NA	NA	(136)
ALKBH5	curcumin	Natural product	CRC	*In vitro*	*In vivo*	(177–179)
	IOX3	Synthetic compound	NA	NA	NA	(180)
	ALK-04	Synthetic compound	melanoma	*In vitro*	*In vivo*	(182)
	compound 3/6	Synthetic compound	AML	*In vitro*	NA	(184)
	MV1035	Synthetic compound	GBM	*In vitro*	*In vivo*	(185, 186)
	compound 20m	Synthetic compound	HCC	*In vitro*	NA	(181)
	Ena15, Ena21	Synthetic compound	GBM	*In vitro*	*In vivo*	(183, 187, 188)
	DDO-2728	Pyrazolo derivative	AML	*In vitro*	*In vivo*	(189–191)
	TD19	Synthetic compound	AML	*In vitro*	NA	(192)
			GBM	*In vitro*	NA	(192)
	W23-1006	Synthetic compound	TNBC	*In vitro*	*In vivo*	(193)
	compound 18	Synthetic compound	AML	*In vitro*	NA	(195)

GBM, Glioblastoma Multiforme; AMOL, Acute Monocytic Leukemia; CC, Cervical Cancer; ccRCC, Clear Cell Renal Cell Carcinoma; GC, Gastric Cancer; EC, Esophageal Cancer; TNBC, Triple-Negative Breast Cancer.

### 5.1 FTO inhibitors without preclinical studies

Aik et al. identified two FTO inhibitors, 2,4-PDCA and IOX1, from the 2-OG analogs (132). Additionally, fluorescein and its derivatives act as bifunctional molecules via selectively inhibiting FTO demethylation while simultaneously labeling FTO proteins. Among fluorescein analogs, FL6 and FL8 perform superior cell permeability and effectiveness in treated cervical cancer HeLa cells (133). N-(5-chloro-2,4-dihydroxyphenyl)-1-phenylcyclobutanecarboxamide (NCDPCB) was identified as another potential FTO inhibitor (134). Similarly, 4-chloro-6-(6′-chloro-7′-hydroxy-2′,4′,4′-trimethyl-chroman-2′-yl) benzene-1,3-diol (CHTB) functions as a small-molecule FTO inhibitor (135). Furthermore, Wang et al. discovered that the natural compound radicicol potently inhibits FTO demethylation in a dose-dependent manner (136). All these inhibitors bind to the same site on FTO as MA. While their cancer inhibitory effects have not yet been fully validated through preclinical studies, these discoveries provide valuable insights into the structure and properties of FTO inhibitors. These foundational knowledges pave the way for the development of more potent, specific, and selective FTO inhibitors with enhanced therapeutic potential.

### 5.2 Natural products of FTO inhibitors

Rhubarbic acid (Rhein), a natural compound, was identified as the first bioactive competitive FTO inhibitor (137). This potent inhibitor with cellular activity binds to FTO’s active site, blocking m6A substrate recognition (137). Therefore, the FTO-m6A axis has emerged as a novel marker characterizing leukemia cell heterogeneity and a broad defense mechanism for its resistance to tyrosine kinase inhibitors (TKIs) (138). And when combined with TKIs, Rhein effectively eradicates drug-resistant leukemia cells and suppresses tumor growth in AML mouse models (138). Mechanistically, Rhein targets FTO to disrupt the AKT/mTOR signaling pathway (139). These findings highlight Rhein’s potential as a therapeutic agent in targeting FTO and overcoming drug resistance in leukemia.

Through suppressing activation of PTEN/PI3K/AKT/mTOR and MAPK/ERK pathways, which are induced by vascular endothelial growth factor and endothelial growth factor, Rhein effectively inhibits the growth of BC cells (140). Additionally, Rhein exhibits antitumor effects on MCF-7 cells with high HER2 expression, mechanistically involved in caspase-9-mediated apoptosis and ROS-mediated activation via NF-kB/P53 signaling pathway (141). Another study further evaluated Rhein’s anti-tumor function on BC cells and tissues through the combination of Rhein and atezolizumab (142). Mechanistically, they remarkably elevate the CD8^+^ T-cell infiltration, serum TNFα and IL-6 levels, apoptotic factors, and the Bax/Bcl2 mRNA level (142). These findings emphasize Rhein’s potential as a complementary agent to enhance the efficacy of immunotherapy in BC.

Saikosaponin-D (SsD), a triterpenoid saponin compound extracted from Bupleurum species, demonstrates significant anti-tumor activity. Sun et al. (143) reported that SsD effectively inhibits AML cells in both *in vitro* and *in vivo* models. Mechanistically, SsD directly targets FTO, leading to an increase in overall RNA m6A methylation levels. This increased methylation level reduces the stability of downstream gene transcripts and inhibits associated signaling pathways, thereby exerting a potent anti-tumor effect. Furthermore, the study uncovered that SsD can overcome FTO/m6A-mediated leukemia resistance to TKIs, making it a promising candidate for addressing drug resistance in AML therapy.

Mupirocin is also a natural FTO inhibitor. Qiao et al. (144) demonstrated that it effectively suppresses CRC cell growth through a ferroptosis-dependent manner. Moreover, FTO promotes the expression of SLC7A11 and GPX4, thereby reducing cancer cell ferroptosis in animal models and *in vitro*. These findings offer valuable insights for the design of novel therapeutic approaches for CRC.

As inhibitors, natural products often lack target specificity, prompting increasing research interest in the exploration of synthetic small-molecule inhibitors.

### 5.3 Synthetic compounds of FTO inhibitors

MO-I-500, a synthetic ascorbic acid analog, has been shown to inhibit FTO activity effectively. FTO exerts a tumor-promoting role in BC. Highly expressed FTO in human BC tissues significantly facilitates tumor progression (145, 146). The mechanism involves Bcl-2 nineteen kilodalton interacting protein 3 (BNIP3), a pro-apoptotic gene (147). FTO decreases m6A modification level of BNIP3 mRNA, resulting in a downregulation of BNIP3 expression. This enables FTO to exert its pro-tumorigenic effects (146). By targeting FTO, MO-I-500 exhibits anti-proliferative effects on BC cells, highlighting its potential as a therapeutic compound in BC treatment (145).

Meclofenamic acid (MA) and its derivative are substrate-competitive inhibitors of FTO with cellular activity. MA is a highly selective inhibitor, and competitively inhibits the binding of FTO to m^6^A-containing nucleic acids (148). Preliminary *in vitro* experiments have demonstrated that MA can inhibit the proliferation of BC, bladder cancer, and NSCLC cells (149). Additionally, BCRP and MRP-7 trigger Gefitinib (GE) resistance during tumor therapy process (150, 151). Therefore, inhibiting BCRP and MRP-7 function or reducing their expression could serve as promising strategies to overcome GE resistance in NSCLC (152). The combination of MA and GE significantly downregulated BCRP and MRP7 expression in GE-resistant cells by increasing m6A modification of MYC (153). This drug combination also inhibits GE resistance by accelerating apoptosis, inhibiting the EGFR downstream pathway, and promoting GE accumulation in cancer cells (153). Furthermore, the application of MA, when combined with TKIs in leukemia management exhibits comparable clinical efficacy to Rhein-based therapies (138). MA2, an acetyl derivative of MA, is another highly selective inhibitor of FTO (148). MA2 significantly inhibits the growth and self-renewal of glioblastoma stem cells (GSCs), thereby suppressing GSCs-induced tumor formation and prolonging the survival time of an animal model (154). MYC/miRNA/MXI1 feedback loop plays a crucial role in glioma proliferation and tumorigenesis. Among them, MYC suppresses MXI1 expression through related miRNA, while MXI1 represses MYC expression via binding to its promoter. Meanwhile, FTO works through stabilizing MYC transcripts. MA2 inhibits FTO activity, thereby disrupting this oncogenic feedback loop and exerting anti-tumor effects. Additionally, MA2 enhances the effectiveness of temozolomide (TMZ) in suppressing glioma cell proliferation (155), highlighting its potential as a therapeutic agent in glioblastoma treatment.

According to previous FTO inhibitors, Prakash et al. utilized a fragment-merging strategy to design and synthesize compound 11b, a hybrid analog combining key fragments of Hz and MA (156). This Hz-MA hybrid analog is a highly effective, selective, and cell-active FTO inhibitor (156). Treatment with Hz-MA in acute monocytic leukemia cells significantly reduces cell viability. Mechanistically, Hz-MA treatment induced upregulation of MYC oncogene expression while simultaneously suppressing RARA transcriptional activity (156). These findings position Hz-MA as a promising candidate for targeted leukemia therapy.

Firstly, two promising FTO inhibitors, FB23 and FB23-2, was designed through structural optimization. These compounds directly bind to FTO and selectively inhibit its m6A demethylase activity (157). FB23, an MA-derived inhibitor, shows more potent inhibition of FTO enzyme activity than MA. And FB23-2, a further optimized analog of FB23, exhibits superior cell permeability. FTO overexpression exerts oncogenic function in AML (158). Thus FB23–2 exerts anti-leukemic effects through multiple mechanisms: inhibiting AML cell proliferation, inducing differentiation and apoptosis, thereby significantly improving survival outcomes in leukemic mouse models (157). Tarullo et al. obtained similar anti-tumor effects against AML (159). However, Wang et al. confirmed the anticancer effect of FB23–2 in cervical cancer, with its mechanism involving the mRNA and protein levels of DIRAS family GTPase 1 (DIRAS1) (160). Additionally, Xu et al. found that FTO is significantly up-regulated in clear cell renal cell carcinoma (ccRCC), where it functions as an oncogene (161). This leads to the reduced level of m6A modification associated with autophagy, and predicts a poor prognosis for patients (161). Mechanistically, the FTO upregulation reduces m6A levels and destabilizes SIK2 mRNA through an m6A-IGF2BP2-dependent pathway, leading to decreased SIK2 expression (161). As a result, ccRCC exhibits enhanced autophagy, proliferation, migration, and invasion. Importantly, FB23–2 inhibits tumor growth to reverse the above malignant processes and prolong survival in animal models (161). Based on previous inhibitors, FB23 analogs (13a and 44/ZLD115) came into light. It was reported that 13a exhibits strong anti-proliferative effects in AML cells and improves survival outcomes in animal models (162). Mechanistically, 13a upregulates ASB2 and RARA expression while downregulating MYC (162). Similarly, 44/ZLD115, a tetracyclic benzoic acid derivative with a flexible basic side chain, significantly upregulates RARA and downregulates MYC to realize its anti-leukemic activity (163). Another promising analog, Dac51, shares a similar FTO-binding mode with FB23 but exhibits greater potency. Dac51 stabilizes FTO while simultaneously blocking FTO-mediated immune evasion, synergizing with ICIs to enhance anti-tumor efficacy (164). Dac51 remodels the tumor microenvironment by attenuating glycolytic metabolism and promoting CD8^+^ T cell infiltration, leading to significant anti-tumor effects in melanoma (164). Additionally, combining Dac51 with ICIs improves therapeutic outcomes (164). These findings exhibit the versatility and potential of FB23-derived inhibitors across a variety of malignancies.

FTO-02 and FTO-04, two potent and highly selective FTO competitive inhibitors, was developed by structural design, synthesis, and biochemical evaluation (165). The research demonstrated that FTO-04 effectively prevented neurosphere growth in patient-derived GSCs, and both the m6A and m6Am levels were elevated in patients after FTO-04 treatment, which ultimately led to the suppression of glioblastoma (165). In another study, through rational design and optimization, a new class of inhibitor oxaprostanes derived from FTO-04 came into birth (166). Among these, FTO-43N emerged as a lead compound, effectively elevating m6A and m6Am levels in gastric cancer cells, ultimately exerting anti-cancer effects by regulating the Wnt/PI3K-Akt signaling pathway (166). These findings highlight the potential of oxaprostanes, particularly FTO-43N, as promising therapeutic drugs for FTO-targeted cancer therapy.

R-2HG, a 2-OG competitive inhibitor, has demonstrated broad antileukemic effects both *in vitro* and *in vivo*. Su et al. (167) showed that R-2HG suppresses the proliferation and survival of leukemia cells while promoting cell cycle arrest and apoptosis. Mechanistically, R-2HG targets the FTO/m6A/MYC/CEBPA signaling pathway to inhibit the malignancy of leukemia cells with high FTO expression. By disrupting this pathway, R-2HG effectively reduces the oncogenic potential of FTO-overexpressing leukemia cells, making it a promising therapeutic agent for leukemia treatment.

CS1 and CS2, potent and selective FTO inhibitors, was identified through a series of screening and validation analyses (168). Studies revealed FTO’s significant role and potential mechanism in prohibiting tumor stem cell self-renewal and immune evasion. CS1 and CS2 selectively bind and occupy the catalytic pocket of FTO, preventing m6A-modified oligonucleotides from entering the catalytic pocket, thereby inhibiting FTO-mRNA binding and reducing MYC/CEBPA while increasing RARA/ASB2 expression (158, 167). Conversely, uninhibited FTO reduced RARA and ASB2 protein expression and suppressed all-trans retinoic acid (ATRA)-induced leukemia cell differentiation (158). Phan et al. found that CS1 inhibits CRC progression through the downregulation of several signaling pathways, including the expression of ERG, KRAP, PDE4B and SLC38A2 (169). In addition, CS1 can be applicable as a single alternative drug or in combination to overcome medicine resistance to 5-FU-based treatments for CRC (169). Moreover, Garg et al. demonstrated that PCa cells express higher levels of FTO than normal cells, and CS1-mediated inhibition of FTO performs significant anti-PCa function (170). These findings underscore the versatility of CS1 and CS2 as promising therapeutic methods against multiple cancers, offering potential for targeted therapy and overcoming drug resistance.

18077 and 18097 was designed to directly inhibit FTO demethylase activity with potency and cellular activity. 18097 has a significant inhibitory effect on the colony formation of BC cells (171). Additionally, 18097 exhibits strong anti-tumor activity *in vivo*, effectively inhibiting the progression of BC and lung metastases (171). Moreover, 18097 was found to metabolically inhibit cancer progression by regulating the key downstream effector cytokine signaling 1(SOCS1) in the P53 pathway (171). This finding makes 18097 a promising candidate for BC therapy targeting FTO.

1,2,3-Triazoles, an important category of nitrogen-containing heterocyclic compounds, possess strong anticancer activity (172). A class of 1,2,3-triazole-pyridine hybrid compounds containing pentafluorobenzoyl groups was designed as FTO inhibitors. Among them, compound C6 showed the strongest inhibitory effect, making it a highly efficient and low-toxicity oral anticancer agent (173). Increased expression of FTO in esophageal cancer tissues is associated with poor clinical prognosis (174). FTO plays an oncogenic role in esophageal cancer, promoting cell proliferation and migration (175). Compound C6 targets FTO to display anti-tumor activity against esophageal cancer via the inhibition of the EMT pathway. Furthermore, C6 regulates the PI3K/AKT signaling pathway, enhancing its inhibitory effect on esophageal cancer progression (173). These findings pave the way for esophageal cancer treatment.

Recently, Liang et al. identified a series of novel and effective FTO inhibitors with an acylhydrazone scaffold, among which compound 8t is a potent representative (176). Compound 8t can inhibit the proliferative activity of AML cells both *in vivo* and *in vitro*, laying the foundation for further research on effective cancer inhibitors.

## 6 ALKBH5 inhibitors

ALKBH5, the second m6A demethylase to be identified, has m6A as its only known catalytic substrate (66). There are few ALKBH5 inhibitors studied to date, especially natural compounds. As a phenolic compound extracted from turmeric root, Curcumin has been shown to reduce the expression of ALKBH5. Then curcumin enhances the translation of tumor necrosis factor receptor-associated factor 4 (TRAF4) and promotes the binding of TRAF4 to the m6A methyl-recognizing enzyme, thereby improving the efficiency of m6A methylation modification (177). Additionally, curcumin facilitates the conversion of microtubule-associated protein LC3-I to LC3-II, upregulates Beclin-1, and induces autophagy in CRC cells. These actions reduce cancer stem cell production and re-sensitize drug-resistant cells to chemotherapeutic agents such as 5-FU and OXA (178, 179). These findings highlight curcumin’s potential as a therapeutic agent targeting ALKBH5-mediated pathways in cancer treatment.

Natural products often lack selectivity, which is why an increasing number of studies are focused on developing selective small molecule inhibitors with targeted competency. Aik et al. unexpectedly discovered that the small molecule inhibitor IOX3 can covalently inhibit ALKBH5 during their study of the structure and function of ALKBH5 (180). Unfortunately, the application of this inhibitor in cancer has not been further explored. Fang et al. (181) obtained an effective compound, 5-hydroxy-1-(3-(trifluoromethyl) phenyl)-1H-pyrazole-3-carboxylic acid (20m), through a series of complex methods. They demonstrated that 20m can inhibit the demethylation process of m6A in HCC HepG2 cells. Additionally, they validated the efficacy, selectivity, and biological activity of 20m as an ALKBH5 inhibitor. However, further *in vivo* and *in vitro* validation is lacking.

ALK-04, a small-molecule inhibitor of ALKBH5, was identified through screening using the X-ray crystal structure and structure-activity relationship of the ALKBH5 protein. In preclinical studies, ALK-04 demonstrated significant therapeutic potential by synergistically reducing melanoma tumor growth when combined with GVAX/anti-PD-1 immunotherapy via MCT4/SLC16A3 axis (182). This finding turns ALK-04 into a promising candidate for enhancing the efficacy of immunotherapy in melanoma treatment.

ALKBH5, which is highly expressed in GSCs, significantly promotes tumor formation and proliferation (183). A study selected two compounds, 2-[(1-hydroxy-2-oxo-2-phenylethyl) sulfanyl] acetic acid (compound 3) and 4- (184)-1,2-diazinane-3,6-dione (compound 6), and validated their antiproliferative activity in various leukemia cell lines (184). All above lay the foundation for the development of selective ALKBH5 inhibitors. Malacrida et al. provided further evidence about MV1035, an ALKBH5-targeted inhibitor (185). They found that MV1035 inhibits the migration and invasion of glioblastoma cells. In a follow-up study, they reaffirmed these findings and demonstrated that MV1035, when combined with TMZ, significantly reduces cell viability and sphere formation in glioblastoma cells (186). Notably, MV1035 not only decreases glioblastoma migration and invasion but also overcomes TMZ resistance, making it a promising candidate for glioblastoma therapy (186). These studies highlight the therapeutic potential of ALKBH5 inhibitors, particularly MV1035, in addressing the challenges of glioblastoma progression and drug resistance.

By high-throughput screening, Takahashi et al. (187) identified two novel small-molecule inhibitors, Ena15 and Ena21, which exhibit non-competitive or competitive inhibition of 2-OG. The tumor-initiating cell GSCs are highly self-renewing, resistant to conventional treatments, and responsible for tumor recurrence because of sustained tumor growth (188). ALKBH5 reduces the m6A methylation level of FOXM1 transcripts, thereby enhancing FOXM1 expression. Where FOXM1 plays a critical role in regulating GSC proliferation, self-renewal, and tumorigenicity (183). Ena15 and Ena21 effectively inhibit cell proliferation in glioblastoma multiforme (GBM) cell lines by downregulating FOXM expression (187). These findings highlight the potential of Ena15 and Ena21 as therapeutic agents targeting ALKBH5-mediated pathways in glioblastoma.

Through structure-based virtual screening and optimization, DDO-2728, a potent and selective pyrazolo[1,5-a] pyrimidine derivative, was identified to directly bind to ALKBH5 (189). Unlike 2-hydroxyglutarate analogs, DDO-2728 selectively inhibits the demethylase activity of ALKBH5 without affecting FTO. Overexpression of ALKBH5 plays a tumorigenic role in AML by regulating the stability of the prognosis-related oncogene TACC3 mRNA (190). DDO-2728 inhibits ALKBH5, resulting in increased m6A modification levels in AML cells, decreased TACC3 mRNA stability, and induction of apoptosis and cell cycle arrest. These antiproliferative effects significantly suppress tumor growth (189). Furthermore, Fei et al. (191) developed and synthesized the covalent ALKBH5 inhibitor DDO-02267 via exploring the protein structure of ALKBH5 and incorporating a salicylaldehyde warhead into a noncovalent small molecule ligand. With the high selectivity and specificity for ALKBH5, DDO-02267 enhances m6A levels and modulates the ALKBH5-AXL signal pathway in AML tissues and cells. These findings highlight the therapeutic potential of ALKBH5 inhibitors in AML.

Recently, researchers developed a selective covalent inhibitor of ALKBH5, TD19, which shows good biological activity (192). And it exhibits significant anticancer efficacy in AML and GBM cells (192). Besides, some other researchers developed a more efficient novel covalent inhibitor, namely W23-1006, which exhibits selectivity and biological activity (193). Experiments about TNBC indicated that W23–1006 covalently inhibits ALKBH5, increasing the m6A level of fibronectin 1 (FN1) mRNA, thereby reducing the expression of FN1 and bringing overall tumor suppression both *in vivo* and *in vivo* (193). Notably, FN1 is a key gene that promotes the EMT process in tumors (194). More recently, investigators identified a series of maleimide-derived small molecule inhibitors targeting ALKBH5, with compound 18 emerging as a key candidate after optimization (195). AML cell assays further confirmed its antiproliferative activity, while comprehensive evaluations highlighted its potential as a lead compound for ALKBH5 inhibitor development (195). These findings offer fresh insights and novel strategies for advancing anticancer therapeutics.

## 7 Inhibitors of IGF2BPs

As is shown in **Table 3**, IGF2BPs share similar structures and functions. Their N-terminal contains two RNA recognition motifs (RRM1-2), while their C-terminal houses four KH homology domains (KH1-4) (196). Notably, IGF2BPs, particularly IGF2BP1 and IGF2BP3, are recognized as oncofetal proteins. These regulatory proteins, which are transiently expressed during embryonic development and subsequently silenced in most adult tissues, undergo tumor-specific reactivation and play pivotal roles in cancer progression and metastasis (33). Aberrant expression of IGF2BPs is associated with a wide range of tumorigenic processes. By regulating the transcripts of several oncogenes such as KRAS, MYC, PTEN, and MDR1, IGF2BPs contribute to numerous malignant behaviors, including enhanced cell proliferation, invasion, stemness, drug-resistance, and immune evasion (196, 197). These properties underline their significance as key players in tumor progress and potential therapeutic targets.

**Table 3:** Summary of m6A methylation recognition protein inhibitors.

Targets	Inhibitors	Types	Cancers	Research types		Ref.
IGF2BP1	BTYNB	Synthetic compound	NSCLC	*In vitro*	*In vivo*	(199)
			melanoma	*In vitro*	NA	(200)
			OC	*In vitro*	*In vivo*	(199, 200)
			ICC	*In vitro*	*In vivo*	(201)
			AML	*In vitro*	NA	(202)
			NENs	*In vitro*	*In vivo*	(203)
			NB	*In vitro*	*In vivo*	(204, 205)
			MM	*In vitro*	*In vivo*	(206)
	Compound 7773	Synthetic compound	OC	*In vitro*	NA	(207)
			NSCLC	*In vitro*	NA	(207)
	AVJ16	Compound 7773 derivative	NSCLC	*In vitro*	NA	(208)
			CRC	*In vitro*	NA	(208)
IGF2BP2	CWI1-2	Synthetic compound	AML	*In vitro*	*In vivo*	(210)
	JX5	Synthetic compound	T-ALL	*In vitro*	NA	(211)
IGF2BP3	Berberine	Natural product	CRC	*In vitro*	*In vivo*	(215, 216)
YTH	Ebselen	Synthetic compound	NA	NA	NA	(217)
	SAC	Synthetic compound	NA	NA	NA	(218)
	compound N-7	Synthetic compound	NA	NA	NA	(219)
	compound 40	Synthetic compound	AML	*In vitro*	NA	(220)

NENs, Neuroendocrine Neoplasms; MM, Multiple Myeloma; T-ALL, T-Cell Acute Lymphoblastic Leukemia.

### 7.1 IGF2BP1 inhibitors

IGF2BP1, also known as ZBP1, VICKZ1, CRD-BP, or IMP1, is an RNA-binding protein with six RNA-binding domains. It plays a key role in tumorigenesis by regulating the translation, stability, localization, and selective splicing of its target mRNAs (198). 2-[(5-bromo-2-thienyl) methylene] amino benzamide (BTYNB) is a structure-specific small molecule inhibitor of IGF2BP1. In OC and NSCLC cell lines and mouse models, BTYNB disrupts E2F-driven gene expression and then controls tumor growth (199). In OC and melanoma cells with high IMP1 expression, BTYNB effectively decreases intracellular c-MYC mRNA and protein levels, thereby suppressing tumor proliferation (200). In ICC cells and tissues, the overexpression of IGF2BP1 promotes growth and inhibits senescence by regulating the c-MYC/p16 axis (201). Additionally, IGF2BP1 induces tumor metastasis by activating the ZIC2/PAK4/AKT/MMP2 axis (201). Fortunately, BTYNB exerts critical antitumor effects in ICC models (201). Furthermore, Jamal et al. showed that BTYNB induces leukemia cell death and cell cycle arrest by upregulating BAK and p21, and promotes differentiation by modulating associated genes such as ITGAM, ZFPM1, and KLF5 (202). Sperling et al. demonstrated that BTYNB suppresses the IGF2BP1/MYC/EZH2 axis’s pro-proliferative effects on neuroendocrine neoplasms by blocking IGF2BP1 binding to MYC mRNA (203). Biegel et al. indicated that BTYNB strengthens neuroblastoma cells’ sensitivity to chemotherapy (204). Hagemann et al. suggested that IGF2BP1/MYCN serves as a poor prognostic factor for high-risk neuroblastoma (205). They also discovered that BTYNB can counter the synergistic pro-carcinogenic effects of IGF2BP1/MYCN by inhibiting IGF2BP1 *in vivo* (205). Xu et al. established a novel IGF2BP1/CDC5L pathogenic axis in multiple myeloma (MM), where BTYNB effectively suppresses the expression of CDC5L, thereby inhibiting MM progression (206). These findings highlight BTYNB as a promising therapeutic agent for IGF2BP1-driven cancers, with broad applicability across divorce cancers.

Compound 7773 was identified as a potent IGF2BP1 inhibitor through FP-based high-throughput screening (HTS) (207). It was shown to directly bind to intracellular IGF2BP1 and block its interaction with target mRNAs, including Kras (207). As a result, Kras mRNA and protein levels were reduced, leading to suppression of pERK signaling (207). Moreover, compound 7773 demonstrated significant inhibitory effects on OC and lung adenocarcinoma cells, suggesting its potential for targeting IGF2BP1-related pathways in various cancers.

AVJ16 was optimized from the lead compound 7773, which was experimentally shown to be a more potent, selective, and safer small-molecule inhibitor of IGF2BP1 (208). AVJ16 exhibits significant anti-cancer effects on NSCLC and CRC cell lines by the downregulation of Kras mRNA and protein (208). Which further confirms its potential as an effective therapeutic agent against cancers with elevated IGF2BP1 expression.

### 7.2 IGF2BP2 inhibitors

IGF2BP2 was validated as a potential anticancer target through various *in vitro* and *in vivo* experiments. KH structural domains, especially the KH3–4 double structural domain, are essential for the binding of IGF2BP2 to m6A-modified RNAs (83). HTS was used to identify 10 potential inhibitors of IGF2BP2/RNA interactions, mainly divided into two categories: benzamidobenzoic acid and ureidothiophene (209). These compounds demonstrated target-specific activity and promising anti-proliferative effects, establishing a framework for developing optimized IGF2BP2 inhibitors with enhanced potency and selectivity against IGF2BP2-driven cancers.

CWI1-2, a potent small molecule inhibitor, was developed to bind directly to IGF2BP2 and competitively inhibit its interaction with target transcripts (210). Elevated IGF2BP2 in AML portends adverse clinical outcomes. IGF2BP2 drives AML progression by m^6^A-dependently controlling glutamine metabolic regulators (MYC/GPT2/SLC1A5), sustaining leukemic stem cell self-renewal (210). Inhibition of IGF2BP2 with CWI1–2 demonstrates favorable antileukemic effects (210). Additionally, combining CWI1–2 with drugs like doxycycline hyclate or homoharringtonine enhances therapeutic efficacy on AML (210). These results position CWI1–2 as a promising therapeutic agent, both alone and in combination therapies targeting IGF2BP2 in AML.

IGF2BP2 is highly expressed in T-cell acute lymphoblastic leukemia (T-ALL), and directly binds to the T-ALL oncogene NOTCH1 in an m6A-dependent manner, thereby stabilizing its mRNA and promoting tumorigenesis (211). Furthermore, IGF2BP2 reduces apoptosis induced by chemotherapeutic agents such as acitretin, vincristine, vannamei, or dexamethasone, contributing to chemoresistance in T-ALL (211). Importantly, the IGF2BP2 inhibitor JX5 not only effectively increases apoptosis but also alleviates chemotherapy resistance, highlighting its potential for treating T-ALL (211).

### 7.3 IGF2BP3 inhibitors

IGF2BP3 acts as an oncogene and a reliable independent prognostic biomarker in CRC (212, 213). IGF2BP3 is markedly overexpressed in colon cancer, where it regulates the cell cycle and angiogenesis by recognizing m6A modifications of target mRNAs, thereby facilitating tumor growth (214). Berberine (BBR), an isoquinoline alkaloid derived from coptis chinensis, exhibits a wide range of pharmacological activities (215). BBR inhibits CRC cell proliferation via the down-regulation of IGF2BP3 (215). Further research demonstrated that BBR targets the degradation of IGF2BP3 through TRIM21-mediated ubiquitination (216). These findings show the therapeutic potential of BBR as an IGF2BP3 inhibitor, offering a promising strategy for treating CRC.

## 8 YTH domain family inhibitors

Though no m6A-recognizing protein inhibitor with antitumor activity has been identified to date. Organoselenium compound ebselen was reported to be a first-in-class inhibitor of the YTHDF m6A-binding domain, which binds and interacts with YTHDF (217). This interaction disrupts the ability of YTHDF recognizing and binding m6A-modified mRNA. Additionally, researchers found a series of structural analogs of ebselen, which are suitable for the development of new inhibitors. Their findings pave the way for developing effective inhibitors targeting m6A-recognizing proteins.

Salvianolic acid C (SAC) was identified as a selective small molecule inhibitor of YTHDF1, exhibiting a competitive inhibitory effect (218). A new pan-inhibitor, N-7, was developed to target YTHDF1-3, YTFDC1, and YTFDC2 (219). Although the *in vitro* inhibitory activity of SAC and N-7 has been confirmed, their application in cancers has not yet been realized. Recently, researchers designed a selective and potent ligand of YTHDC1, known as compound 40, which demonstrates significant anti-proliferative activity across various AML cell lines (220). All above lay the foundation for further refinement and promotion of potent inhibitors.

## 9 Therapeutic promise, clinical limitations, and future perspectives of m6A regulatory protein inhibitors

m6A regulatory factor inhibitors, as emerging anti-tumor agents targeting RNA epigenetic modifications, exhibit multiple therapeutic advantages in oncology. First, their high selectivity enables precise targeting of cancer cells, enhancing treatment accuracy (193, 221). Second, due to their target specificity, most inhibitors demonstrate minimal toxicity to critical organs such as the heart, liver, and kidneys at effective doses, resulting in a relatively broad therapeutic window (222). Third, by disrupting the dynamic balance of oncogenic RNAs, these inhibitors open novel avenues for investigating cancer-related molecular mechanisms (223, 224). Fourth, m6A-associated inhibitors have been shown to remodel immune cell functions and alter the tumor microenvironment, thereby potentiating anti-tumor immune responses (225–228). Fifth, they can overcome resistance to conventional therapies and exhibit synergistic effects when used in combination regimens (223, 229, 230).

Nonetheless, the application of m6A-related inhibitors also presents certain limitations. Foremost among them is the context-dependent function of m6A regulatory proteins, which may exhibit diametrically opposing roles in different tumor types—leading to unpredictable therapeutic outcomes or even pro-tumorigenic effects. For instance, ALKBH5 functions as an oncogene in TNBC but acts as a tumor suppressor in gastric cancer; thus, its inhibition may suppress TNBC metastasis while potentially promoting GC progression (193, 231). Moreover, beyond their roles in tumorigenesis, m6A regulatory proteins are intricately involved in physiological processes such as hematopoiesis and neurogenesis (232). Insufficient target specificity, or off-target effects among protein family members, may impair normal cellular function and cause toxicity concerns. For example, though YTHDF1 inhibition showed anti-leukemic activity, it also impaired the self-renewal capacity of normal hematopoietic stem cells (229). In addition, m6A regulatory factor inhibitors face similar physicochemical limitations as other small-molecule agents, including poor aqueous solubility, metabolic instability, and inadequate cell permeability, which collectively hinder the potential of transferring into drugs (223, 233). Finally, due to mechanistic complexity and the involvement of non-enzymatic functions, single-agent inhibition is sometimes insufficient to fully suppress oncogenic signaling pathways (18).

To address these challenges, researchers are actively exploring next-generation drug design strategies. These include enhancing selectivity, improving drug delivery systems, and identifying rational combination therapies. For selectivity, dual-target inhibitors (e.g., FTO/ALKBH5 inhibitors) and proteolysis-targeting chimeras (PROTACs) degradation technology have been proposed as potential solutions to increase specificity (48). In terms of drug delivery, researchers aim to optimize drug delivery systems to overcome pharmacokinetic limitations (93). Tan et al. (234) designed a hydrogel loaded with STM2457, which adheres to the surgical margins of CRC patients when injected. The hydrogel slowly and continuously releases the inhibitor, inhibiting METTL3 to suppress m6A methylation in CRC. This inhibition leads to increased expression of CXCL9 and CXCL10, effectively recruiting infused CAR-NK cells to prevent postoperative recurrence of CRC. Li et al. (235) developed a novel nano-delivery system, PLGA-STM-TAT, consisting of PLGA (poly lactic-co-glycolic acid) nanoparticles loaded with the METTL3 inhibitor STM2457 and the cell-penetrating peptide TAT. This nanoparticle-loaded drugs can modulate m6A methylation and target EphA2 through selectively accumulating in gastric cancer tissues. The system not only significantly enhances drug delivery but also inhibits cancer cell proliferation by downregulating key oncogenes, c-MYC and BRD4. For combination strategies, investigators seek to explore novel combination therapies to maximize efficacy and overcome resistance. Chen et al. (225) innovatively combined the FTO inhibitor CS2 with the anti-PD-1 body for the treatment of HCC, demonstrating favorable efficacy in both *in vitro* and *in vivo* experiments. Mechanistically, glycoprotein non-metastatic melanoma protein B (GPNMB), a downstream target of FTO, serves as an oncogenic factor in HCC. Tumor cell-derived extracellular vesicles encapsulate GPNMB and deliver it to surface-associated receptors on CD8+ T cells, thereby suppressing their immune activity. However, CS2, by targeting the FTO/m6A/GPNMB axis, significantly enhances the therapeutic efficacy of anti-PD-1 body. This study provides insight into the potential combination of m6A modification-related inhibitors with ICIs for improved therapeutic strategies.

**Figure 2:**
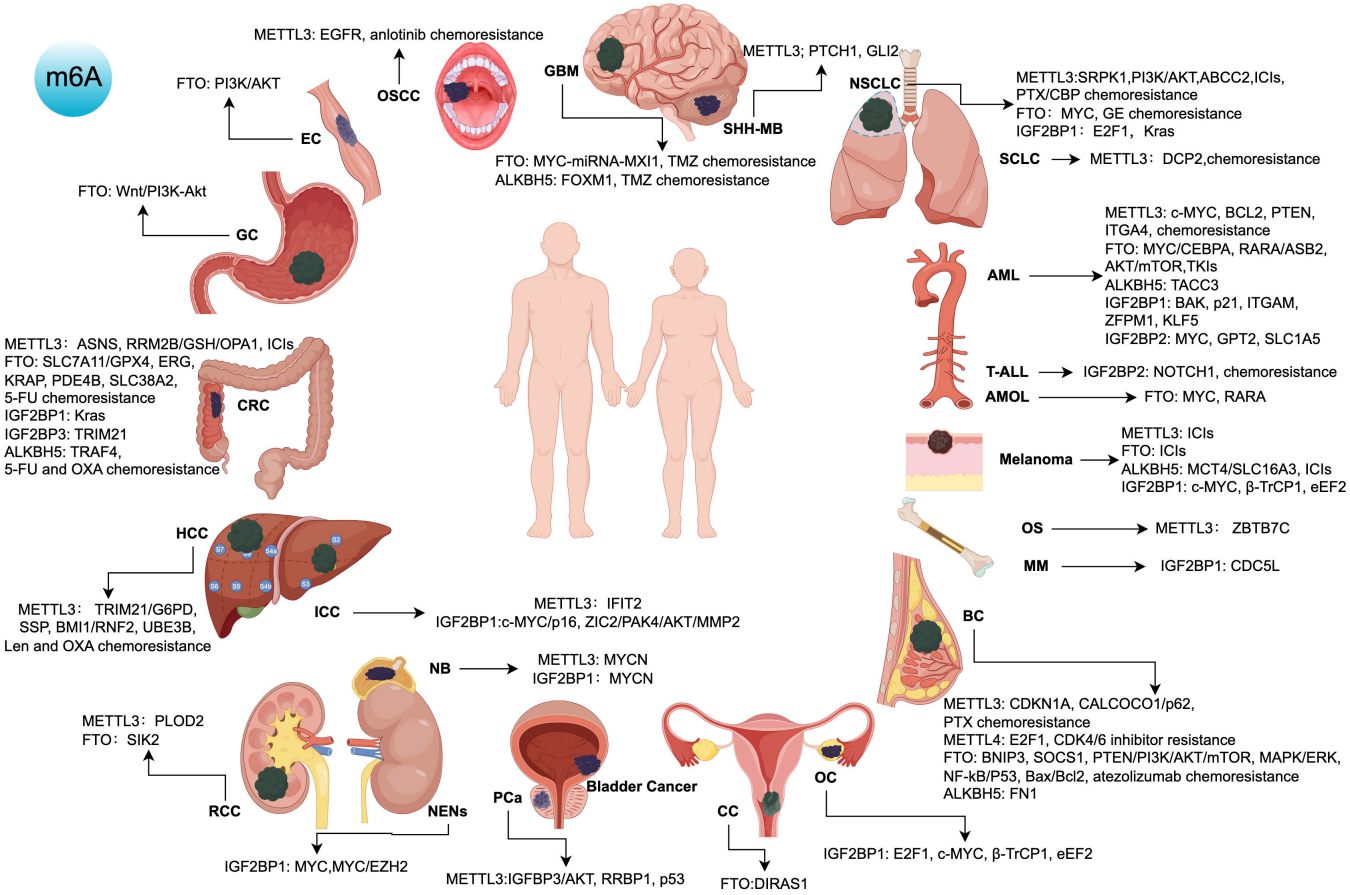
Targets, signaling pathways, and roles of m6A regulatory protein inhibitors in various cancers. ICIs, immune checkpoint inhibitors; TKIs, tyrosine kinase inhibitors; 5-FU, 5-Fluorouracil; OXA, Oxaliplatin; Len, Lenvatinib; PTX, Paclitaxel; CBP, Carboplatin; GE, Gefitinib; TMZ, Temozolomide; AML, Acute Myeloid Leukemia; PCa, Prostate Cancer; HCC, Hepatocellular Carcinoma; OS, Osteosarcoma; NSCLC, Non-Small Cell Lung Cancer; SCLC, Small Cell Lung Cancer; ICC, Intrahepatic Cholangiocarcinoma; RCC, Renal Cell Carcinoma; NB, Neuroblastoma; SHH-MB, Sonic Hedgehog Medulloblastoma; CRC, Colorectal Cancer; OSCC, Oral Squamous Cell Carcinoma; BC, Breast Cancer; OC, Ovarian Cancer; GBM, Glioblastoma Multiforme; AMOL, Acute Monocytic Leukemia; CC, Cervical Cancer; GC, Gastric Cancer; EC, Esophageal Cancer; NENs, Neuroendocrine Neoplasms; MM, Multiple Myeloma; T-ALL, T-Cell Acute Lymphoblastic Leukemia.

Based on the previous inhibitor targets, as shown in **Figure 2** (by Figdraw), these innovative approaches integrating m6A-associated inhibitors provide novel therapeutic strategies for cancers prone to residual disease, metastasis, and recurrence, representing a promising direction for future cancer treatment development.

Despite the formidable hurdles, m6A-associated inhibitors remain a promising frontier in cancer therapy owing to their unique mechanisms—such as modulating oncogenic RNA stability, reshaping the immune microenvironment, and altering metabolic reprogramming. Notably, the clinical translation of m6A regulatory factor inhibitors remains to face substantial obstacles. Though a growing body of preclinical research supports their potential, only a few candidates have advanced into clinical trials. Issues such as potential toxicity to normal cells, insufficient understanding of resistance mechanisms, and a lack of reliable biomarkers to identify responsive patient populations all contribute to the hurdles facing clinical development. These gaps also highlight critical directions for future investigations aimed at optimizing m6A-targeted therapies.

## 10 Conclusion

In conclusion, although m6A regulatory protein inhibitors exhibit a duality of therapeutic potential and clinical risks, m6A and its regulatory proteins remain a prominent research focus, particularly in oncology. With advancing research, m6A regulators have become increasingly recognized as therapeutic targets across various cancers. Targeted inhibition of m6A regulatory proteins demonstrates promising therapeutic potential in suppressing tumor growth, enhancing chemosensitivity, and promoting cancer immunotherapy. In this review, we systematically summarize all reported inhibitors targeting m6A regulators, providing comparative analyses of their antitumor mechanisms and efficacy across various cancers. Additionally, we critically evaluate their complementary roles in overcoming chemotherapy resistance and enhancing immunotherapy effect. Notably, this review highlights innovative therapeutic approaches utilizing novel drug delivery systems for these inhibitors. Additionally, this review summarizes next-generation drug design strategies for m6A-associated inhibitors and their clinical translation challenges. These findings establish a foundation for developing novel cancer therapies and bring good news to patients with currently incurable malignancies.

## Glossary

METTL5: Methyltransferase-like 5
METTL16: Methyltransferase-like 16
RBM15: RNA Binding Motif Protein 15
RBM15B: RNA Binding Motif Protein 15B
ZC3H13: Zinc Finger CCCH-Type Containing 13
ZCCHC4: Zinc Finger CCHC-Type Containing 4
CBLL1: Cbl Proto-Oncogene Like 1
VIRMA: Vir Like m6A Methyltransferase Associated
HNRNPs: Heterogeneous Nuclear Ribonucleoproteins
eIF3: Eukaryotic Translation Initiation Factor 3
G3BP2: GTPase-Activating Protein SH3 Domain-Binding Protein 2
PRRC2A: Proline-Rich Coiled-Coil 2A
RBMX: RNA Binding Motif Protein X-Linked
FMR1: Fragile X Messenger Ribonucleoprotein 1
ELAVL1: ELAV Like RNA Binding Protein 1
G3BP1: GTPase-Activating Protein SH3 Domain-Binding Protein 1
IMP1: Insulin-like Growth Factor 2 mRNA-Binding Protein 1
IMP2: Insulin-like Growth Factor 2 mRNA-Binding Protein 2
IMP3: Insulin-like Growth Factor 2 mRNA-Binding Protein 3
RRBP1: Ribosome Binding Protein 1
c-MYC: MYC Proto-Oncogene
BCL2: B-Cell Lymphoma 2
PTEN: Phosphatase and Tensin Homolog
SP1: Specificity Protein 1
SPP2: Secreted Phosphoprotein 2
ITGA4: Integrin Subunit Alpha 4
SRPK1: Serine/Arginine-Rich Protein-Specific Kinase 1
PI3K: Phosphatidylinositol 3-Kinase
AKT: Serine/Threonine Kinase
ABCC2: ATP-Binding Cassette Subfamily C Member 2
DCP2: mRNA Decapping Enzyme 2
IFIT2: Interferon-Induced Protein with Tetratricopeptide Repeats 2
TRIM21: Tripartite Motif-Containing Protein 21
G6PD: Glucose-6-Phosphate Dehydrogenase
PSPH: Phosphoserine Phosphatase
PSAT1: Phosphoserine Aminotransferase 1
PHGDH: Phosphoglycerate Dehydrogenase
BMI1: B Lymphoma Mo-MLV Insertion Region 1 Homolog
RNF2: Ring Finger Protein 2
HIF-1α: Hypoxia-Inducible Factor 1 Subunit Alpha
PLOD2: Procollagen-Lysine,2-Oxoglutarate 5-Dioxygenase 2
KAP1: KRAB Associated Protein 1
MYCN: MYCN Proto-Oncogene
PTCH1: Patched 1
GLI2: GLI Family Zinc Finger 2
ZBTB7C: Zinc Finger and BTB Domain Containing 7C
RRM2B: Ribonucleotide Reductase Regulatory TP53 Inducible Subunit M2B
OPA1: Mitochondrial Dynamin Like GTPase
POP1: Processing of Precursors 1
CDKN1A: Cyclin Dependent Kinase Inhibitor 1A
XBP1s: X-Box Binding Protein 1 Spliced
CALCOCO1: Calcium Binding and Coiled-Coil Domain 1
mTOR: Mechanistic Target of Rapamycin Kinase
MAPK: Mitogen-Activated Protein Kinase
ERK: Extracellular Signal-Regulated Kinase
NF-kB: Nuclear Factor Kappa B
P53: Tumor Protein P53
Bax: BCL2 Associated X
SLC7A11: Solute Carrier Family 7 Member 11
GPX4: Glutathione Peroxidase 4
BCRP: ATP Binding Cassette Subfamily G Member 2
MRP-7: Multidrug Resistance Protein Mrp-7
MYC: Proto-Oncogene
EGFR: Epidermal Growth Factor Receptor
MXI1: MAX Interactor 1
RARA: Retinoic Acid Receptor Alpha
DIRAS1: DIRAS Family GTPase 1
SIK2: Salt Inducible Kinase 2
ASB2: Ankyrin Repeat and SOCS Box Containing 2
CEBPA: CCAAT Enhancer Binding Protein Alpha
ERG: ETS Transcription Factor ERG
KRAP: SUGP1 (SURP And G-Patch Domain Containing 1)
PDE4B: Phosphodiesterase 4B
SLC38A2: Solute Carrier Family 38 Member 2
TRAF4: TNF Receptor Associated Factor 4
LC3-I: Microtubule Associated Protein 1 Light Chain 3
LC3-II: Microtubule Associated Protein 1 Light Chain 3
MCT4: Solute Carrier Family 16 Member 3
FOXM1: Forkhead Box M1
TACC3: Transforming Acidic Coiled-Coil Containing Protein 3
FN1: Fibronectin 1
KRAS: Proto-Oncogene
MDR1: ATP Binding Cassette Subfamily B Member 1
ZBP1: Z-DNA Binding Protein 1
VICKZ1: IGF2BP1 (Insulin Like Growth Factor 2 mRNA Binding Protein 1)
CRD-BP: IGF2BP1 (Insulin Like Growth Factor 2 mRNA Binding Protein 1)
p16: Cyclin Dependent Kinase Inhibitor 2A
ZIC2: Zic Family Member 2
PAK4: p21-Activated Kinase 4
MMP2: Matrix Metallopeptidase 2
BAK: BCL2 Antagonist/Killer 1
ITGAM: Integrin Subunit Alpha M
ZFPM1: Zinc Finger Protein FOG Family Member 1
KLF5: Kruppel Like Factor 5
IGF2BP1: Insulin Like Growth Factor 2 mRNA Binding Protein 1
IGF2BP2: Insulin Like Growth Factor 2 mRNA Binding Protein 2
IGF2BP3: Insulin Like Growth Factor 2 mRNA Binding Protein 3
EZH2: Enhancer of Zeste 2 Polycomb Repressive Complex 2 Subunit
CDC5L: Cell Division Cycle 5 Like
GPT2: Glutamic–Pyruvic Transaminase 2
SLC1A5: Solute Carrier Family 1 Member 5
NOTCH1: Notch Receptor 1
CXCL9: C-X-C Motif Chemokine Ligand 9
CXCL10: C-X-C Motif Chemokine Ligand 10
BRD4: Bromodomain Containing 4
GPNMB: Glycoprotein Nmb
